# An Endogenous Peptide Preparation from Oyster Soft Tissues Modulates the Cytoskeletal Gene Expression Programme of Human Intestinal Epithelial Cells

**DOI:** 10.3390/foods15213775

**Published:** 2026-10-22

**Authors:** Jing Zhang, Wenhui Zhou, Lujie Wei, Xi Liu, Luxi Chen, Jianwen Mao, Jingwei Yu

**Affiliations:** 1 School of Life Sciences and Biopharmaceutics, Guangdong Pharmaceutical University, Guangzhou 510006, China; 2 School of Basic Medical Sciences, Guangdong Pharmaceutical University, Guangzhou 510006, China; 3 College of Medical Information Engineering, Guangdong Pharmaceutical University, Guangzhou 510006, China; 4 Key Laboratory of Glucolipid Metabolic Diseases, Ministry of Education, Guangdong Pharmaceutical University, Guangzhou 510006, China

**Keywords:** endogenous peptides, anionic peptides, oyster, intestinal epithelium, Caco-2, cytoskeleton, MRTF–*SRF*

## Abstract

The oyster *Crassostrea hongkongensis* is eaten raw and whole, so the endogenous peptides carried by its tissue reach the intestinal lumen unprocessed; functional work on food-derived peptides has concentrated on cationic sequences. This study asked whether the endogenous peptide population of this species is anionically skewed and whether a preparation of that population alters the cytoskeletal gene programme of human intestinal epithelial cells. The published plasma peptidome of this species (PXD025247) was re-analysed, an endogenous peptide fraction below 10 kDa was prepared from oyster soft tissues without adding any protease, and the preparation was applied to differentiated Caco-2 monolayers at 0.1–2.0 mg/mL for 6–24 h; nine transcripts were measured by quantitative PCR against vehicle, protease-digested and digestion-only controls. Of 333 peptides, 157 (47.1%) carried a net charge of −1 or lower at pH 7, 148 (44.4%) were near-neutral and 28 (8.4%) carried +2 or above, and anionic peptides outnumbered cationic peptides in all four detection strata (36.4–58.3% against 4.8–9.1%). Actin-family proteins were the largest precursor source (186 peptides, 55.9%; 68.3% coverage of actin). The preparation was recovered at 2.41 ± 0.23 mg/g of wet tissues and, at 2.0 mg/mL, increased transcription of the *SRF* targets *CCN1* (2.91-fold), *VIL1* (2.29-fold), *MYH9* (2.17-fold) and *ACTN1* (1.83-fold) (*p* < 0.01 for each) and of *TMSB4X*, *CFL1* and *PFN1* (1.90-, 1.75- and 1.55-fold; q < 0.05), whereas *SRF* and *MRTFA* transcripts were unchanged; proteinase K digestion abolished the response. An endogenous peptide preparation from oyster soft tissues therefore acts on the cytoskeletal gene programme of human intestinal epithelial cells, and the anionically skewed majority of this peptidome is the fraction that warrants direct functional characterisation.

## 1. Introduction

The intestinal epithelium is the first host compartment that dietary proteins and the peptides derived from it meet, and it is the interface at which diet-derived peptides initiate their effects on the host [1,2]. The monolayer combines a selectively permeable barrier with an active sensing system, and its response to luminal material involves the coordinated transcription of genes encoding junctional and cytoskeletal components [3,4]. The structural basis of that response is well defined: the zonula occludens proteins ZO-1, ZO-2 and ZO-3 scaffold the tight junction [5,6], claudins form the sealing strands [4,7] and occludins contribute to junctional strand organisation [8]. The luminal peptide pool that this system encounters is chemically diverse. Peptides released from food proteins during processing, storage and gastrointestinal digestion carry antioxidant, antihypertensive, immunomodulatory, antimicrobial and mineral-binding activities, and several have entered functional-food development [9,10,11,12,13,14,15]. Recent work has extended the same logic to peptide fractions of marine and plant origin, including collagen peptides that protect the intestinal barrier in ulcerative colitis [16] and gluten-derived glutamine peptides that suppress lipopolysaccharide-induced inflammation [17]. The great majority of these preparations are hydrolysates, in which peptide bonds are cleaved by an added protease, so the population studied is defined by the specificity of the enzyme chosen rather than by the food itself [2,11,18]. The luminal contents that the epithelium integrates also include microbial metabolites such as the short-chain fatty acids, whose effects on epithelial and immune gene expression are well described [19]; peptides of dietary origin act on the same cell population through a different chemical route.

Peptides that a food organism synthesises or accumulates in its own tissues constitute a separate class, because they are generated by the organism’s own proteolytic system and are ingested as such when the tissue is eaten [20]. Mass-spectrometry-based peptidomics has mapped these populations across taxa, including the neuropeptidomes of crustaceans, in which endogenous peptides act as signalling molecules [20,21]. Bivalve molluscs are a favourable system in which to examine the class. Oysters are eaten raw in many coastal regions, the soft tissue is consumed whole, and no protease is added at any stage before ingestion, so the endogenous peptides of the tissue reach the intestinal lumen intact [22]. Preparations derived from oysters have been reported to protect the intestine of mice against oxidative damage [23], to attenuate tumour necrosis factor-α-induced inflammation in Caco-2 monolayers [24], to protect hepatocytes in vitro [18] and to improve mineral bioavailability [25]. Peptide discovery in this species has been extended from plasma to the mucus proteome [26] and reviewed for marine invertebrates generally [27]. Endogenous, non-hydrolysate peptide populations of oyster tissues have not been tested against an intestinal epithelial gene programme.

Selection in this field has consistently favoured short, cationic sequences. Cationic and amphipathic peptides are the classical antimicrobial chemotype, they are enriched efficiently by cation-exchange chromatography from hydrolysates, and they are the sequences that antimicrobial peptide databases are built to collect [28,29,30,31]. The same bias is less well justified for activities other than direct bacterial killing. Anionic peptides that carry a net negative charge at physiological pH include the acidic lipopeptide antibiotic laspartomycin [32] and the scorpion venom peptide Ev37 [33]; non-canonical anionic sequences translocate epithelial and endothelial barriers [34]; and acidic peptides act as metal-binding ligands [35]. Charge therefore defines what is selected for study rather than what is present in the food.

Re-analysis of the published plasma peptidome of *Crassostrea hongkongensis* [22] revealed how much a charge-first strategy leaves behind. Of the 333 peptides in the analysis set, 157 (47.1%) carry a net charge of −1 or lower at pH 7 and only 28 (8.4%) carry +2 or above. The original study selected six up-regulated, highly cationic peptides for antimicrobial testing and validated one of them as an antimicrobial peptide, so the functional data available for this dataset describe the 8.4% cationic minority [22]. The acidic character of the remaining peptides is compositional rather than incidental: they contain a median of three acidic residues, 13.6% of their length, and 74.2% of them have a negative grand average of hydropathicity. The skew is also stable with respect to the physiological state of the animal, holding at 43.8% to 47.6% anionic and 4.8% to 9.1% cationic whether the peptides are considered as a whole, restricted to those detected in unchallenged oysters, or restricted to those detected only after bacterial challenge. The anionic majority is therefore a constitutive property of this peptidome and defines the fraction that functional work has not examined, which is the starting point of the present study.

Actin-family proteins are the largest single precursor source of this peptidome, and two independent lines of evidence make the epithelial cytoskeleton a reasonable first target for a population of this kind. First, peptides act directly on actin systems. Chemotactic peptides change F-actin content and its distribution in neutrophils within seconds [36,37]; short proline-rich motifs bind the actin regulators that nucleate filaments [38]; peptide-receptor microclusters organise actin at the plasma membrane [39]; and actin-derived peptides-termed actinators modulate cytoskeleton-related cellular activities through actin-binding proteins [40]. Second, the epithelial cytoskeleton is a transcriptionally regulated structure. Junctional proteins, villin-based apical architecture, myosin-II contractility and actin-crosslinking proteins are all products of a gene programme that is remodelled during inflammation, repair and epithelial plasticity [3,41,42]. The actin-severing protein cofilin-1 regulates intestinal junctional integrity and mucosal inflammation [41], and profilin-1 and thymosin β4 control the monomeric actin pool that feeds filament assembly [43].

The transcriptional node that reads actin dynamics is the myocardin-related transcription factor–serum response factor (MRTF–*SRF*) axis. Monomeric actin retains MRTF cofactors in the cytoplasm; when the filament-to-monomer balance shifts, MRTFs enter the nucleus and activate *SRF*-dependent genes, among the genes encoding actin itself, non-muscle myosin heavy chains, α-actinin, villin and the immediate-early matricellular protein *CCN1* [44,45,46,47,48]. *CCN1* is a canonical immediate-early *SRF* target whose induction requires MRTF and *SRF* activity [49,50]. The two MRTF cofactors, MRTF-A and MRTF-B, are both required for normal development and both potentiate *SRF* activity [51,52]. The axis operates in the intestinal epithelium, where MRTF-A dosage controls homeostatic renewal [53], and its readout is transcriptional rather than post-translational, because regulation occurs at the level of cofactor localisation rather than of *SRF* abundance [44,45]. Epithelial cells sense their mechanical and adhesive environment through integrin-linked actin coupling [54,55], so the axis is positioned to convert a change in luminal chemistry into a change in cytoskeletal gene expression.

In this study, we re-analysed the published plasma peptidome of *C. hongkongensis* to define the charge profile and precursor origin of the endogenous peptides that the animal produces, and we prepared an endogenous peptide fraction from oyster soft tissues without adding any protease. We applied the preparation to differentiated human intestinal epithelial Caco-2 monolayers across 0.1 to 2.0 mg/mL and 6 to 24 h, and measured the nine transcripts that constitute the *SRF*-dependent cytoskeletal programme together with the transcripts encoding the actin-monomer-binding proteins and the core of the pathway. A proteinase K-digested preparation and a digestion-only control established the peptide dependence of the response. We tested two hypotheses: first, that the endogenous peptide population of this species is anionically skewed and that the skew is a constitutive property of the peptidome rather than a feature of a challenge-responsive subset, and second, that a preparation of that population changes the transcription of the *SRF*-dependent cytoskeletal gene programme in differentiated human intestinal epithelial cells. The MRTF–*SRF* axis provides the mechanistic framework for the second hypothesis, and the experiments reported here measure transcript abundance, so they test the transcriptional signature that this axis predicts and do not by themselves establish the upstream steps of the pathway.

## 2. Materials and Methods

### 2.1. Re-Analysis of the Published Oyster Plasma Peptidome

Peptide identifications and label-free intensities were taken from the MaxQuant output deposited with ProteomeXchange dataset PXD025247 [22]. No new mass spectrometry was performed. The peptide table contained 344 rows; entries assigned to the reverse database were removed, leaving the 338 peptides reported by the original authors, and five further entries matching the contaminant database were then removed, giving an analysis set of 333 peptides used for every statistic reported here.

Net charge at pH 7 was calculated as (K + R + 0.1 × H) − (D + E), histidine being weighted at 0.1 to approximate its partial protonation at neutral pH. Monoisotopic peptide masses were calculated from residue masses with the addition of water. The grand average of hydropathicity (GRAVY) was calculated with the Kyte–Doolittle scale. Precursor attribution used the MaxQuant ‘Leading razor protein’ and ‘Proteins’ fields. An entry was counted as actin by sequence rather than by name: the reference proteome deposited with the dataset (17,609 entries) was searched for sequences of 350–400 residues containing the actin signature motif DNGSGMCKAGFAGDDAPR, which identified five entries (EVMG000194.1, EVMG003590.1, EVMG007312.1, EVMG007341.1 and EVMG018240.1). Peptides were counted as actin-derived under three rules: leading razor protein equal to EVMG003590.1; leading razor protein within the actin family; and any actin-family entry present in the protein group. Coverage of actin was computed from the MaxQuant start and end positions of peptides assigned to actin-family entries, relative to the 360-residue EVMG003590.1 sequence.

Peptides were stratified by detection across the six deposited samples, three unchallenged (phosphate-buffered saline) and three bacterially challenged, using non-zero label-free intensity as the criterion for detection. The sensitivity of the challenge-responsive subset to the stringency of the criterion was examined across fifteen combinations of the minimum number of challenged samples (2–6) and the maximum number of unchallenged samples (0–2) in which a peptide was allowed to be detected. Compositional comparisons between charge subsets used the two-sided Mann–Whitney U test and Fisher’s exact test.

### 2.2. Oysters

Oysters (*Crassostrea hongkongensis*) were obtained from a commercial shellfish farm in Maoming, China, in June, 2026, and transported to the laboratory on ice within 3 h of harvest. Live animals were rinsed and opened, and the whole soft body, that is every tissue other than the shell, was dissected out. Three independent preparations were made, each from a separate collection of 20 animals; the tissue was pooled within a preparation before homogenisation.

### 2.3. Preparation of the Endogenous Peptide Fraction

The pooled soft body was homogenised at 4 °C in five volumes (*w*/*v*) of 50 mM Tris-HCl (pH 7.4) containing 150 mM NaCl, 5 mM EDTA and a broad-spectrum protease inhibitor cocktail. No exogenous protease was added at any stage of the preparation. The homogenate was centrifuged at 20,000× *g* for 30 min at 4 °C and the supernatant filtered through a 0.45 µm cellulose acetate membrane. The filtrate was passed through a regenerated cellulose ultrafiltration device with a 10 kDa nominal molecular-weight cut-off (Amicon Ultra-15, 10 kDa NMWCO, Merck Millipore, Darmstadt, Germany) by centrifugation at 4000× *g* at 4 °C until the retentate volume was reduced to one tenth of the starting volume; the permeate was retained. Low-molecular-weight solutes were then removed from the permeate by three cycles of ten-fold dilution and re-concentration against phosphate-buffered saline (PBS, pH 7.4) in a 3 kDa device, so that the material applied to cells contained the endogenous peptide population and PBS rather than the homogenisation buffer. The desalted permeate was lyophilised, reconstituted in PBS at 50 mg/mL, the concentration at which oyster peptide preparations were handled for cell work [18], sterilised through a 0.22 µm polyethersulfone filter and stored at −80 °C in single-use aliquots; no aliquot was subjected to more than one freeze–thaw cycle. The stock was added to serum-free medium at 0.2%, 1.0% and 4.0% (*v*/*v*) to give the working concentrations of 0.1, 0.5 and 2.0 mg/mL, the range over which oyster and marine peptide preparations act on intestinal epithelial cells [16,18,24].

Total peptide content was determined by bicinchoninic acid assay against a bovine serum albumin standard, with three technical replicates per batch. Size distribution was determined by sequential ultrafiltration: the 10 kDa permeate was fractionated on a 3 kDa device under the conditions above, and the peptide content of the <3 kDa and 3–10 kDa fractions was measured by bicinchoninic acid assay and expressed as a percentage of total recovered peptide. Residual protein was assessed by Tricine SDS-PAGE (16.5% separating gel) with densitometric quantification and expressed per milligramme of peptide. Endotoxin was measured with a kinetic turbidimetric Limulus amebocyte lysate assay against a standard curve with a positive product control in duplicate wells; batches exceeding 0.1 EU/µg peptide were to be re-processed by polymyxin B affinity chromatography before use, and every batch used for cell work was below that threshold. A vehicle control consisting of PBS carried through the identical ultrafiltration, desalting and lyophilisation train, but without tissue, was prepared alongside each batch. The bicinchoninic acid assay was used as a total-peptide measure because it is the standard method for this material, and its limitations were taken into account when the values were interpreted: the colour yield of a peptide mixture depends on composition and on chain length, small peptides and glycine-rich sequences give a lower response per unit mass than the bovine serum albumin standard, and the values reported here are therefore relative rather than absolute. Every batch was quantified against the same standard curve under identical conditions, so the values are directly comparable between batches and support the between-batch reproducibility that is reported.

### 2.4. Cell Culture

Caco-2 cells (ATCC HTB-37) were obtained from the Cell Bank of the Chinese Academy of Sciences (Shanghai, China; catalogue no. TCHU 66) and were used between passages 25 and 35. The line is the standard in vitro model of the human intestinal epithelium: it forms a polarised monolayer with tight junctions, brush-border hydrolases and a permeability profile that correlates with human oral absorption [56,57,58,59], and co-culture variants of it have been developed for absorption studies [60]. The line was authenticated by short tandem repeat profiling and confirmed free of mycoplasma contamination before the study and again at the end of it. Cells were maintained inhigh-glucose Dulbecco’s modified Eagle’s medium (Gibco, Grand Island, NY, USA) supplemented with 10% foetal bovine serum, 1% non-essential amino acids and 1% penicillin–streptomycin at 37 °C under 5% CO_2_. For experiments, cells were seeded at 1 × 10^5^ cells/cm^2^ on polyester Transwell inserts (12 mm diameter, 0.4 µm pore; Millipore, Burlington, MA, USA) and allowed to differentiate for 21 days after reaching confluence, with medium renewal every second day. Barrier formation was monitored by transepithelial electrical resistance (TEER) measured with a chopstick electrode on days 0, 7, 14 and 21 after confluence and immediately before treatment. Readings were taken after 20 min equilibration at room temperature with blank-insert subtraction and three readings per insert; monolayers were accepted for treatment at above 350 Ω·cm^2^, the value that confirms barrier formation in this model [61,62]. Barrier integrity was therefore verified in every experiment immediately before treatment. Barrier function was monitored before treatment rather than after it, and the cytoskeletal readout of this study is transcriptional.

### 2.5. Treatment

Differentiated monolayers were washed twice with serum-free medium and incubated with the peptide preparation at 0.1, 0.5 and 2.0 mg/mL in serum-free medium for 6, 12 or 24 h. Vehicle controls received an equal volume of the PBS vehicle that had been carried through the full preparation train. For the protease control, the preparation was digested with proteinase K (20 µg/mL, 2 h, 37 °C; Thermo Fisher Scientific, Waltham, MA, USA) and the enzyme inactivated at 95 °C for 10 min; completeness of digestion was confirmed by Tricine SDS-PAGE, which showed no Coomassie-reactive material above 1 kDa. A digestion-only control containing proteinase K at the same concentration in the vehicle, heat-inactivated identically, was run in parallel. For challenge experiments, monolayers were exposed to lipopolysaccharide (1 µg/mL) with or without the preparation.Cell viability was measured with CCK-8 reagent (Beyotime Biotechnology, Shanghai, China).

Wells were allocated to treatment groups with a randomisation table. The operator performing RNA extraction and quantitative PCR was blinded to group allocation until after the Cq values had been exported. Each of the three independent experiments used a different cell passage and a different batch of the peptide preparation.

### 2.6. RNA Extraction and Quantitative PCR

Total RNA was extracted with TRIzol reagent and treated with DNase I (both Thermo Fisher Scientific, Waltham, MA, USA) and quantified spectrophotometrically; the A260/A280 ratio was required to be above 1.9. Complementary DNA was synthesised from 1 µg of RNA with a reverse transcription kit using random hexamers. Quantitative PCR was performed on a Roche LightCycler 480 II instrument (Roche Diagnostics, Basel, Switzerland) with SYBR Green chemistry (Thermo Fisher Scientific, Waltham, MA, USA) in a final volume of 20 µL with three technical replicates per sample. Cycling was 95 °C for 30 s followed by 40 cycles of 95 °C for 5 s and 60 °C for 30 s, with melt curves acquired from 65 to 95 °C. Primer sequences are given in Table 1; all primers were designed in silico against RefSeq templates, checked for specificity against the human RefSeq RNA collection and validated empirically by standard-curve and melt-curve analysis before use.

Standard curves were generated from five ten-fold serial dilutions of pooled cDNA, and amplification efficiency was calculated as (10^(−1/slope) − 1) × 100. Efficiency between 90% and 110%, a coefficient of determination above 0.99, a single melt peak and no amplification in the no-template control below a Cq of 38 were required for a primer pair to be accepted (Figure S3). Reference-gene stability was assessed with geNorm across a candidate panel comprising *B2M*, *HPRT1*, *RPLP0*, *GAPDH* and *ACTB* [63]. *B2M* and *HPRT1* were selected on the basis of M values below 0.5 and a pairwise variation below 0.15 (Figure S2); *GAPDH* and *ACTB* were excluded because their transcript levels were affected by the treatments, an effect consistent with the sensitivity of *GAPDH* to oxidative stress and of *ACTB* to perturbation of the cytoskeleton. Relative expression was calculated by the ΔΔCq method and reported in accordance with the MIQE guidelines [64].

### 2.7. Statistical Analysis

Each experiment was performed on three independent occasions, and *n* refers to independent experiments throughout; data are presented as mean ± standard deviation. *MYH9*, *ACTN1*, *VIL1* and *CCN1* were pre-specified as primary endpoints and were tested individually against vehicle by one-way analysis of variance followed by Dunnett’s test, without correction for multiplicity because the endpoints were fixed before the experiments began. *CFL1*, *PFN1*, *TMSB4X*, *SRF* and *MRTFA* were analysed as a secondary family, and the Benjamini–Hochberg procedure was applied within that family with a false-discovery rate of 0.05; both the nominal *p* value and the adjusted q value are reported. *p* < 0.05 was considered significant for primary endpoints and q < 0.05 for secondary endpoints. Analyses were performed in GraphPad Prism 9.0 (GraphPad Software, San Diego, CA, USA). Effect sizes are reported with 95% confidence intervals, which were computed for each transcript from the mean, the standard deviation and the three independent experiments as mean ± t_0.975,2_ × SEM.

## 3. Results

### 3.1. Charge Profile of the Oyster Plasma Peptidome

**Figure 1:**
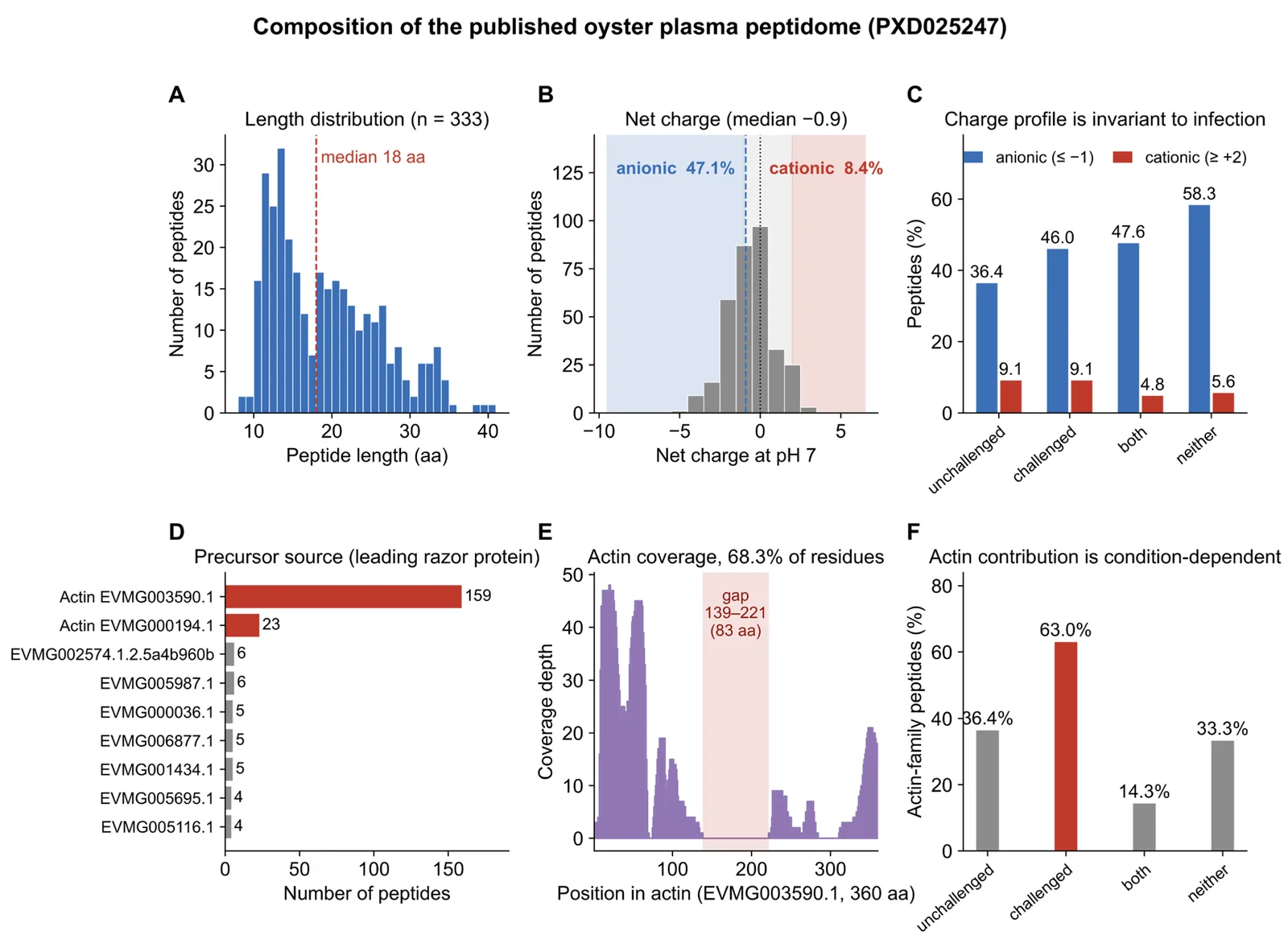
Composition of the published oyster plasma peptidome (PXD025247). (**A**) Length distribution of the 333 peptides in the analysis set (median of 18 residues). (**B**) Distribution of net charge at pH 7 (median of −0.9); the dashed line marks the median and the dotted line neutrality; the anionic (≤−1), near-neutral and cationic (≥+2) classes are shaded, and the proportion in each of the two extreme classes is given. (**C**) Charge profile of the four mutually exclusive detection strata: detected only in unchallenged samples (*n* = 11), detected only after challenge (*n* = 265), detected in both (*n* = 21), and carrying no non-zero intensity in any of the six deposited runs (*n* = 36). The four strata sum to the 333 peptides of the analysis set. Anionic peptides outnumber cationic peptides in every stratum. (**D**) Number of peptides per leading razor protein; actin-family entries are red and every other entry grey. (**E**) Coverage of the actin sequence (EVMG003590.1, 360 residues) by the 186 actin-family peptides; the shaded region marks positions 139–221, the single stretch spanned by no peptide. (**F**) Actin-family contribution to the same four detection strata as (**C**), which is condition-dependent in contrast to the charge profile.

Re-analysis of the peptide table deposited with PXD025247 revealed 344 entries. Removal of reverse-database hits left the 338 peptides reported by the original authors, and removal of five further entries matching the contaminant database gave an analysis set of 333 peptides, which is the set used throughout this work. The peptides ranged from eight to 40 residues with a median of 18, and from 0.93 to 4.38 kDa with a median of 1.95 kDa (Figure 1A).

The charge distribution of this population was strongly asymmetric (Figure 1B). The median net charge at pH 7 was −0.9. Of the 333 peptides, 157 (47.1%) carried a net charge of −1 or lower, 148 (44.4%) laid between −1 and +2, and 28 (8.4%) carried +2 or above. The acidic character is compositional: the median peptide contained three acidic residues, 13.6% of its length, and 74.2% of the peptides had a negative GRAVY value. Anionic peptides therefore constitute roughly half of the population, and the cationic subset that has been the object of functional study is less than one tenth of it.

### 3.2. Charge Profile Across Detection Strata

Because the deposited dataset compares unchallenged and bacterially challenged oysters, the possibility that the charge profile is a property of a challenge-responsive subset was examined directly (Figure 1C). The 333 peptides partition into four mutually exclusive strata, where 11 were detected only in unchallenged samples, 265 only in challenged samples, 21 in both, and 36 carried no non-zero label-free intensity in any of the six deposited runs. The anionic proportion was 36.4%, 46.0%, 47.6% and 58.3% in the four strata respectively, against a cationic proportion of 9.1%, 9.1%, 4.8% and 5.6%; median net charge was zero, −0.8, −0.9 and −1.0. Anionic peptides therefore outnumbered cationic peptides in every stratum, by at least four-fold. The 11-peptide unchallenged-only stratum is the smallest of the four and carries the widest confidence interval of the set.

**Figure 2:**
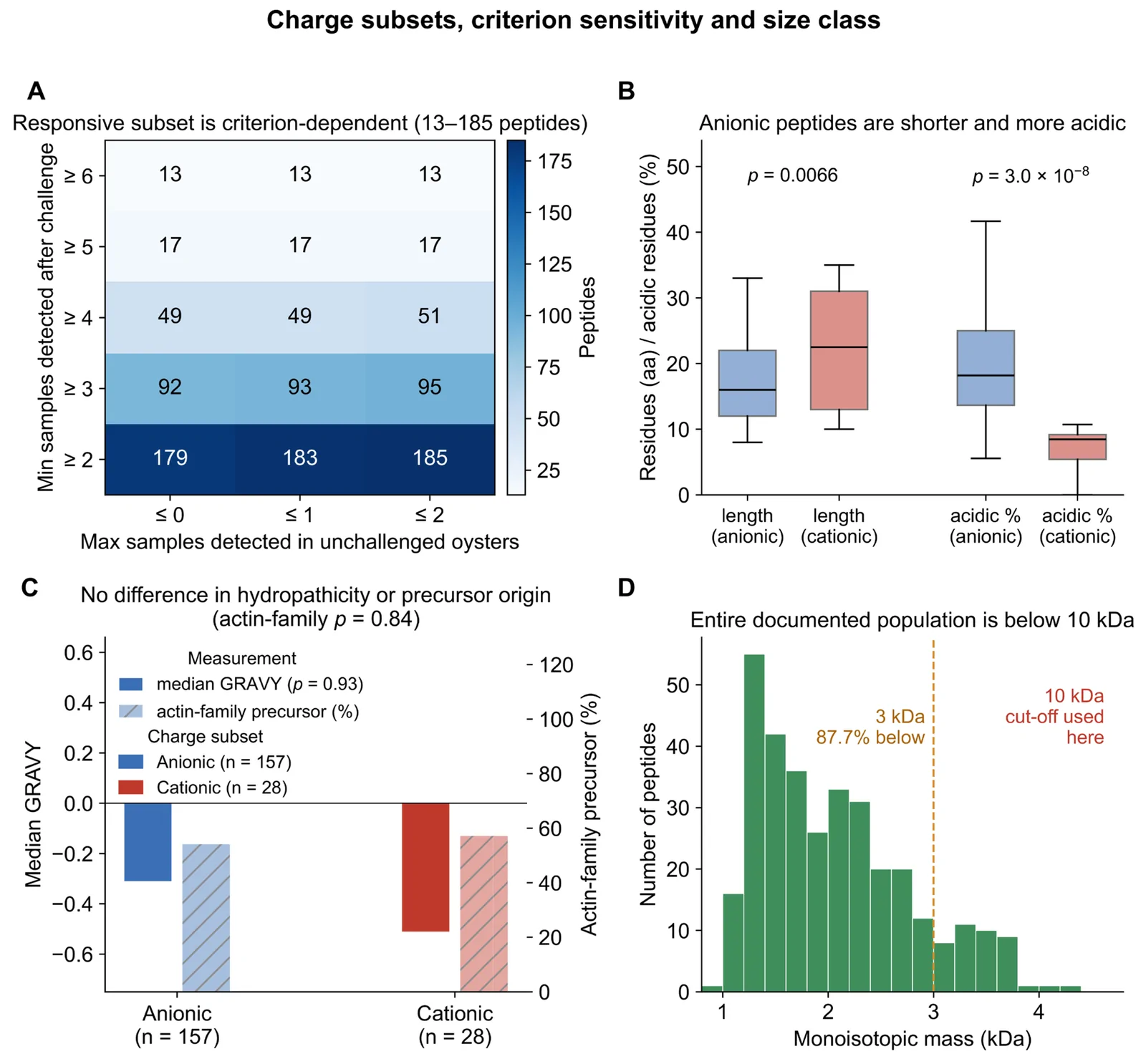
Charge subsets, criterion sensitivity and size class. (**A**) Sensitivity of the challenge-responsive subset to the stringency of the detection criterion; each cell gives the number of peptides satisfying the combination of the minimum number of challenged samples (rows) and the maximum number of unchallenged samples (columns) in which a peptide was allowed to be detected. (**B**) Length and acidic-residue content of the anionic (*n* = 157) and cationic (*n* = 28) subsets; boxes show median and interquartile range with whiskers at 1.5 times the interquartile range, and *p* values are from two-sided Mann–Whitney U tests. (**C**) Median GRAVY (left axis) and actin-family precursor contribution (right axis) of the two subsets; neither differs between them. (**D**) Monoisotopic mass distribution of the 333 peptides with the 3 kDa and 10 kDa cut-offs marked; the entire documented population lies below 10 kDa. In (**B**,**C**),blue denotes the anionic subset and red the cationic subset.

The size of the challenge-responsive subset is itself criterion-dependent. Across fifteen combinations of the minimum number of challenged samples and the maximum number of unchallenged samples in which a peptide was allowed to be detected, the responsive subset ranged from 13 to 185 peptides (Figure 2A). For that reason the compositional quantities reported here are those of the whole 333-peptide population and of the detection-stratified subsets, which do not depend on a threshold choice, rather than of a responsive subset selected by one particular threshold.

### 3.3. Precursor Origin of the Peptidome

We searched the 17,609 protein entries deposited with the dataset for sequences of 350–400 residues containing the actin signature motif DNGSGMCKAGFAGDDAPR and identified five actin-family entries. Under the three counting rules described in the Materials and Methods Section, 159 peptides (47.7%) had EVMG003590.1 as their leading razor protein and 186 peptides (55.9%) were assigned to the actin family; the counts obtained from the leading razor protein and from the protein group were identical. Actin-family proteins were accordingly the largest single precursor source, ahead of every other family in the dataset (Figure 1D).

The 186 actin-family peptides covered 246 of the 360 residues of EVMG003590.1 (68.3%), with a maximum coverage depth of 48 peptides at a single position. Coverage was continuous except for a single gap between positions 139 and 221, an 83-residue stretch that no identified peptide spanned (Figure 1E).

The contribution of actin-family precursors depends on physiological state in a way that the charge profile does not. The proportion was 36.4% among the peptides detected only without challenge, 63.0% among those detected only after challenge, 14.3% among those detected in both and 33.3% among those with no non-zero intensity in any run (Figure 1F). Actin dominance is therefore a property of the challenge-remodelled peptidome, whereas the anionic charge profile is a property of the peptidome as a whole.

### 3.4. Composition of the Anionic and Cationic Subsets

The two charge subsets were compared directly (Figure 2B,C). Anionic peptides (net charge ≤ −1, *n* = 157) were shorter than cationic peptides (≥+2, *n* = 28), with median lengths of 16 and 22.5 residues (*p* = 0.0066) and median masses of 1778 and 2434 Da (*p* = 0.0048). They contained a higher proportion of acidic residues, 18.2% against 8.5% in length (*p* = 3.0 × 10^−8^), and fewer basic residues, with a median of two against four (*p* = 7.4 × 10^−15^). The two subsets did not differ in hydropathicity, with median GRAVY values of −0.31 and −0.51 (*p* = 0.93), and they did not differ in precursor origin: actin-family proteins accounted for 54.1% of the anionic subset and 57.1% of the cationic subset (*p* = 0.84). Charge therefore partitions this peptidome by length and by acidic-residue content, not by the protein family from which the peptides are liberated. The twenty most abundant members of the anionic class, ranked by label-free intensity in the challenged samples, with their sequences, masses, hydropathicity values and precursor assignments, are listed in Table 2.

### 3.5. Size Class and Yield of the Tissue Preparation

Every peptide in the analysis set laid below the 10 kDa cut-off used for the experimental preparation: the largest was 4.38 kDa and 292 of the 333 peptides (87.7%) were below 3 kDa (Figure 2D). A 10 kDa ultrafiltration therefore retains the entire size class documented in the plasma peptidome of this species, and a 3 kDa cut-off would exclude just over one tenth of it.

Three independent preparations from oyster soft tissues gave a total peptide yield of 2.41 ± 0.23 mg per gram of wet tissues, with a between-batch coefficient of variation of 9.4% (Table 3, Figure S1). The <3 kDa and 3–10 kDa fractions accounted for 71.2 ± 2.3% and 28.8 ± 2.3% of recovered peptide respectively. Residual protein was between 18.9 and 26.8 µg per milligramme of peptide, and endotoxin content ranged from 0.038 to 0.061 EU/µg, below the 0.1 EU/µg threshold applied to every batch used for cell work. The tissue preparation is therefore enriched in the same size class as the documented plasma population, where 71.2% of the tissue preparation and 87.7% of the plasma population lie below 3 kDa.

### 3.6. Concentration–Response of the SRF Target Genes

**Figure 3:**
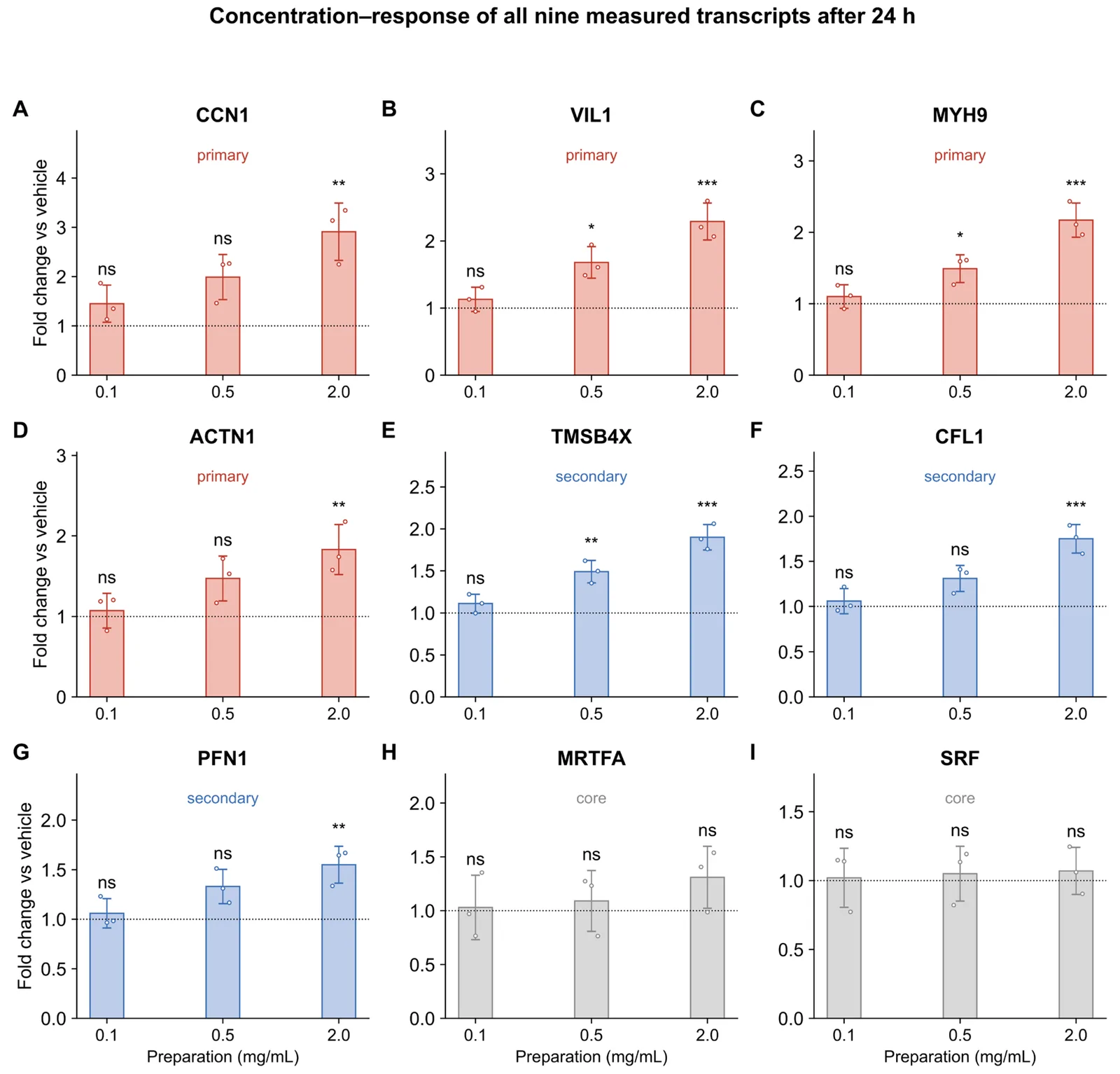
Concentration–response of all nine measured transcripts after 24 h of exposure to the endogenous peptide preparation. (**A**–**D**) Pre-specified primary endpoints *CCN1* (**A**), *VIL1* (**B**), *MYH9* (**C**) and *ACTN1* (**D**). (**E**–**G**) Transcripts encoding the actin-monomer-binding proteins *TMSB4X* (**E**), *CFL1* (**F**) and *PFN1* (**G**). (**H**,**I**) Core components of the pathway, *MRTFA* (**H**) and *SRF* (**I**). Bars show mean ± SD of three independent experiments with individual values overlaid; the vehicle group is 1.00 by definition. Significance markers are from one-way analysis of variance with Dunnett’s test against vehicle. Primary endpoints (**A**–**D**) were pre-specified; secondary endpoints (**E**–**I**) were assessed with Benjamini–Hochberg correction within the secondary family, and both the nominal *p* values and the adjusted q values are given in Table 4. Asterisks indicate significance against vehicle assessed by one-way analysis of variance with Dunnett’s test (* *p* < 0.05; ** *p* < 0.01; *** *p* < 0.001); ns, not significant.

**Figure 4:**
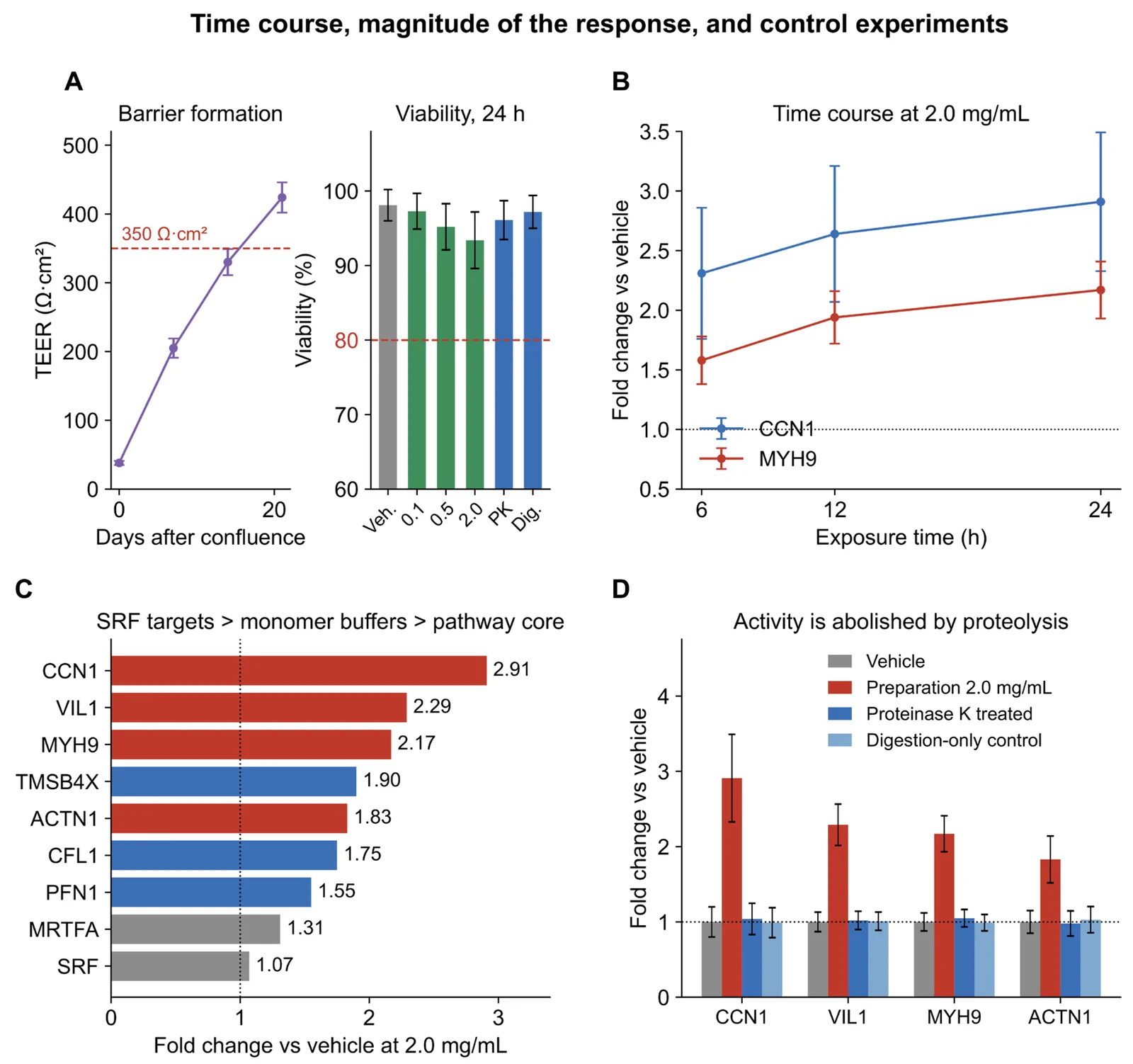
Time course, magnitude of the response, and control experiments. (**A**) Transepithelial electrical resistance before treatment and cell viability after 24 h. (**B**) Time course of *CCN1* and *MYH9* expression at 2.0 mg/mL. (**C**) Fold change at 2.0 mg/mL for all nine transcripts, ordered by magnitude: *CCN1* responded most strongly, the structural *SRF* targets *VIL1* and *MYH9* followed, *TMSB4X*, *ACTN1*, *CFL1* and *PFN1* lay in the range 1.55- to 1.90-fold, and the pathway core responded least. (**D**) Effect of pre-digesting the preparation with proteinase K on the four primary endpoints, with the vehicle and the digestion-only control shown alongside. All panels show mean ± SD of three independent experiments.

The measurements reported in this section are transcript abundances. They define a transcriptional response and do not by themselves establish a change in cytoskeletal protein abundance, in filament organisation or in barrier function. Monolayers reached a TEER above 350 Ω·cm^2^ by day 21 after confluence and were treated at that point.In our experiments, exposure to the peptide preparation changed the expression of all four pre-specified primary endpoint genes in a concentration-dependent manner (Figure 3A–D). At 2.0 mg/mL, *CCN1* expression increased 2.91-fold (*p* = 0.0014 versus vehicle; one-way analysis of variance with Dunnett’s test), *VIL1* 2.29-fold (*p* = 0.0001), *MYH9* 2.17-fold (*p* < 0.0001) and *ACTN1* 1.83-fold (*p* = 0.0083), and all four endpoints reached significance. At 0.5 mg/mL the increases were smaller (1.99-, 1.68-, 1.49- and 1.47-fold respectively), and *VIL1* (*p* = 0.0111) and *MYH9* (*p* = 0.0289) reached significance while *CCN1* laid at the margin (*p* = 0.0532). No transcript differed from vehicle at 0.1 mg/mL (*p* > 0.4 for all four). The response was present at 6 h and had not declined by 24 h (Figure 4B). Cell viability remained above 92% at the highest concentration tested (Figure 4A).

**Table 4:** Effect of the endogenous peptide preparation (2.0 mg/mL, 24 h) on the nine measured transcripts in differentiated Caco-2 monolayers.

Gene	Role	Fold Change vs. Vehicle	SD	95% CI of Fold Change	Vehicle SD	*p* Value (Dunnett)	q Value (BH)	Decision
*CCN1*	Primary (*SRF* target)	2.91	0.582	1.46–4.36	0.2	0.0014	—	Significant
*VIL1*	Primary (*SRF* target)	2.29	0.275	1.61–2.97	0.13	0.0001	—	Significant
*MYH9*	Primary (*SRF* target)	2.17	0.239	1.58–2.76	0.12	<0.0001	—	Significant
*ACTN1*	Primary (*SRF* target)	1.83	0.311	1.06–2.60	0.15	0.0083	—	Significant
*TMSB4X*	Secondary (actin-binding)	1.9	0.152	1.52–2.28	0.08	<0.0001	<0.0001	Significant
*CFL1*	Secondary (actin-binding)	1.75	0.158	1.36–2.14	0.1	0.0003	0.0007	Significant
*PFN1*	Secondary (actin-binding)	1.55	0.186	1.09–2.01	0.1	0.0062	0.0103	Significant
*MRTFA*	Secondary (pathway core)	1.31	0.288	0.59–2.03	0.23	0.4251	0.5314	Not significant
*SRF*	Secondary (pathway core)	1.07	0.171	0.65–1.49	0.17	0.9405	0.9405	Not significant

Values are the mean of three independent experiments, each using a different cell passage and a different batch of the preparation. The vehicle group is expressed as 1.00 by definition of the ΔΔCq calculation. Its standard deviation, propagated from the ΔCq values of the three vehicle samples, is given per gene in the column headed ‘Vehicle SD’. Effect sizes are given with their 95% confidence intervals, computed from the mean, the standard deviation and the three independent experiments as mean ± t_0.975,2_ × SEM. Coefficients of variation at 2.0 mg/mL range from 8.0% (*TMSB4X*) to 22.0% (*MRTFA*); track transcript abundance: the transcripts measured at the lowest Cq (*TMSB4X*, *CFL1*, *PFN1*; Table S1) were the most reproducible and those measured at the highest Cq (*MRTFA*, *SRF*, *CCN1*) the least. Primary endpoints were pre-specified and tested individually against vehicle by one-way analysis of variance with Dunnett’s test; no multiplicity correction was applied because the endpoints were fixed before the experiments began. Secondary endpoints were analysed as one family and the Benjamini–Hochberg procedure was applied with a false-discovery rate of 0.05. *TMSB4X*, *CFL1* and *PFN1* remained significant after correction; *MRTFA* and *SRF* did not. The response is concentration-dependent: no transcript differed from vehicle at 0.1 mg/mL, the two structural endpoints *VIL1* and *MYH9* reached significance at 0.5 mg/mL, and all four primary endpoints did so at 2.0 mg/mL. The full concentration–response data are shown in Figure 3. Cell viability was above 92% in every group at 24 h, and transepithelial electrical resistance was above 350 Ω·cm^2^ immediately before treatment in every experiment.

### 3.7. Actin-Monomer-Binding Transcripts and Pathway Core

We measured the transcripts encoding actin-binding proteins and the core components of the pathway in the same samples (Figure 3E–I). At 2.0 mg/mL, *TMSB4X* increased 1.90-fold (*p* < 0.0001, q < 0.0001), *CFL1* 1.75-fold (*p* = 0.0003, q = 0.0007) and *PFN1* 1.55-fold (*p* = 0.0062, q = 0.0103); all three remained significant after Benjamini–Hochberg correction within the secondary family. *MRTFA* showed a smaller increase that did not reach significance (1.31-fold, *p* = 0.4251, q = 0.5314), and *SRF* transcript levels were unchanged (1.07-fold, *p* = 0.9405). Seven transcripts moved in the same direction, with *CCN1* responding most strongly (2.91-fold), the structural *SRF* targets *VIL1* and *MYH9* next (2.29- and 2.17-fold), and *TMSB4X*, *ACTN1*, *CFL1* and *PFN1* together in the range 1.55- to 1.90-fold (Figure 4C).

### 3.8. Peptide Dependence of the Response

Our protease control showed that pre-digestion of the preparation with proteinase K abolished the changes in *MYH9*, *ACTN1*, *VIL1* and *CCN1* expression (Figure 4D). Expression in the protease-treated group did not differ from vehicle (1.04-, 0.98-, 1.06- and 1.02-fold; *p* > 0.05 for all four genes), and the digestion-only control containing heat-inactivated proteinase K in vehicle was likewise indistinguishable from vehicle (*p* > 0.05). The vehicle was carried through the identical ultrafiltration, desalting and lyophilisation train, and the digestion-only control excludes an effect of the enzyme or of its heat-inactivated products.

## 4. Discussion

### 4.1. Principal Findings

Utilising the published plasma peptidome of *C. hongkongensis* together with an endogenous peptide preparation from oyster soft tissues, this study reports two connected findings. First, the endogenous peptide population of this species is anionically skewed, and the skew is invariant to the physiological state of the animal. Second, an endogenous peptide preparation from oyster soft tissues, which contains no added protease, changes the cytoskeletal gene programme of differentiated human intestinal epithelial cells. At 2.0 mg/mL the preparation increased transcription of the four pre-specified *SRF* targets, most strongly for *CCN1*, and of the three transcripts encoding actin-monomer-binding proteins, while leaving *SRF* itself unchanged. The response was concentration-dependent, present within 6 h, and abolished by digestion of the preparation with proteinase K. The activity resides in the endogenous peptide component of the preparation, and the anionically skewed majority of the peptidome is the fraction that warrants direct functional characterisation.

### 4.2. The Anionic Majority of a Food Peptidome

The literature on food-derived peptides has been built predominantly on cationic and amphipathic sequences, for reasons that are partly chemical and partly technical [28,29,31]. Cationic peptides are the classical antimicrobial chemotype [28,29], they are collected systematically by the antimicrobial peptide databases that the field uses as reference [30], and they are enriched conveniently by cation exchange from hydrolysates [11]. The same preference is visible in oyster research, where antimicrobial peptide discovery has moved from the plasma peptidome [22] to the mucus proteome with machine-learning screening [26] and has been reviewed as the principal peptide activity of marine invertebrates [27]. Our analysis quantifies what that preference costs in this species: the cationic subset that has been characterised is 8.4% of the peptidome, and the 47.1% of peptides carrying a net charge of −1 or lower have not been tested.

Anionic peptides are active in several settings unrelated to direct bacterial killing. The acidic lipopeptide antibiotic laspartomycin is active against Gram-positive pathogens [32]; the anionic scorpion peptide Ev37 restricts viral entry by altering endosomal pH [33]; non-canonical anionic sequences cross epithelial and endothelial barriers [34]; and acidic peptides coordinate metal ions [35]. Anionic character also determines behaviour in the gut lumen. Peptides with a low isoelectric point resist the anionic endopeptidases that dominate intestinal proteolysis and remain intact at acidic pH, which favours their survival to the epithelial surface [65]. The 44.4% of the population that is near-neutral at pH 7 constitutes a third class that remains equally uncharacterised.

### 4.3. Relationship to Previous Work on Oyster and Other Food Peptides

The comparison with previous work on oyster-derived material is instructive in three respects. First, the starting material differs. Reported preparations are hydrolysates produced with added proteases [18] or aqueous extracts of whole tissues [24], so their peptide content is defined by an added enzyme or by endogenous autolysis during extraction. The preparation used here contains no added protease, and the homogenisation buffer was exchanged for PBS before use, so the peptides are those the animal’s own proteolytic system had produced at the time of harvest.

Second, the biological endpoint differs. Oyster peptides derived from *Crassostrea gigas* protected the mouse intestine against cyclophosphamide-induced oxidative damage through Nrf2–Keap1 signalling [23], and an oyster soft-tissue extract attenuated tumour necrosis factor-α-induced inflammation in Caco-2 cells [24]. Both endpoints are inflammatory or redox-related. The endpoint reported here is the cytoskeletal gene programme, a distinct arm of the epithelial response that defines the structural and junctional framework within which the redox and inflammatory responses operate. Oyster protein hydrolysates have been characterised for molecular-weight distribution and antioxidant activity in hepatocytes [18], and oyster peptide–ferrous chelates have been developed to improve iron bioavailability [25]; both studies measured biochemical rather than transcriptional endpoints.

Third, the same logic has been applied successfully to peptides from other foods in the intestinal setting. Marine collagen peptides from fish scales protect the intestinal barrier and modulate redox status in ulcerative colitis [16]; a glutamine peptide released stepwise from corn gluten meal alleviates lipopolysaccharide-induced inflammation [17]; casein hydrolysates strengthen the intestinal barrier [66]; yeast hydrolysates attenuate lipopolysaccharide-induced intestinal inflammation [67]; and casein peptides have been reviewed as regulators of inflammatory bowel disease from an intestinal-barrier perspective [68]. Food peptides also assemble into supramolecular materials that accumulate in inflamed colon tissue [69]. Consistent with our findings, it has been reported that food-derived peptides act on the intestinal epithelium at the level of barrier and junctional gene expression, and every one of these populations was generated by an added protease or by fermentation. Our results extend that conclusion to a peptide population that no added enzyme produced, and to a charge class that selection has discarded.

### 4.4. Magnitude and Pattern of the Transcriptional Response

The response is graded rather than uniform, and the ordering carries mechanistic information. *CCN1*, an immediate-early *SRF* target that is induced within an hour of a change in actin dynamics and that requires both MRTF and *SRF* activity [49,50], responded most strongly at every concentration tested, reaching 2.91-fold at 2.0 mg/mL. The structural *SRF* targets encoding villin, non-muscle myosin heavy chains and α-actinin followed, at 2.29-, 2.17- and 1.83-fold. The transcripts encoding the actin-monomer-binding proteins *TMSB4X*, *CFL1* and *PFN1* responded over the same range, at 1.90-, 1.75- and 1.55-fold, and the pathway core responded least: *MRTFA* rose 1.31-fold and *SRF* did not change, neither reaching significance. This ordering is the signature of a transcription factor–target relationship. An immediate-early target reports the activity of the pathway directly, whereas transcripts encoding structural and monomer-buffering proteins accumulate more slowly and are additionally subject to feedback, and a change in the activity of a pathway does not require a change in the transcript level of its own components. The unchanged transcript levels of *SRF* and *MRTFA* place regulation of the axis at the level of cofactor localisation rather than of transcript abundance [44,45].

The magnitudes match those reported for other food-derived peptide preparations acting on intestinal cells, where changes of roughly 1.5- to 3-fold in transcript or protein readouts are typical [16,17,23,24], and they are smaller than the changes produced by pharmacological activation of the pathway [70,71]. A 2.91-fold change in *CCN1* and a 2.29-fold change in *VIL1* in a polarised human epithelial monolayer, produced by a preparation that contains no added protease and no purified compound, are substantial effects on a food-derived material. The fact that *SRF* transcript levels are unchanged while seven downstream transcripts move in the same direction is a stronger signal than any single fold change considered alone.

### 4.5. Mechanistic Interpretation

The pattern of change is consistent with an effect on actin dynamics that is transmitted to transcription through MRTF–*SRF* signalling. In epithelial cells the proportion of monomeric actin is sensed by MRTF cofactors, whose nuclear accumulation drives *SRF*-dependent expression of genes encoding cytoskeletal and junctional proteins [44,45,46]. The coordinated change in *CCN1*, *VIL1*, *MYH9* and *ACTN1*, together with parallel changes in *TMSB4X*, *CFL1* and *PFN1* transcripts, is the pattern that this axis would be expected to produce when the filament-to-monomer balance is perturbed, and the contemporaneous change in the transcripts encoding actin-monomer-binding proteins points to the monomer pool as the likely site of action. Cofilin-1 severs filaments and increases the turnover of the polymer [72,73,74] and its severing activity is switched off by LIM-kinase-dependent phosphorylation [75]; the pathway as a whole drives directed cell movement [76]; profilin-1 delivers monomers to growing barbed ends [43]; and thymosin β4 sequesters monomers [43]. The simultaneous rise in all three transcripts is consistent with a coordinated adjustment of the machinery that sets the filament-to-monomer ratio.

The concurrent rise in the three monomer-binding transcripts and in *SRF* target transcription could be mediated through a perturbation upstream of the monomer pool, at Rho-family signalling or at integrin-linked adhesion, both of which converge on MRTF nuclear import [45,54,55]. Peptides engage actin systems directly: chemotactic peptides change F-actin content in neutrophils within seconds [36,37], proline-rich motifs bind the actin-nucleating machinery [38], and actin-derived actinators modulate cytoskeleton-related activities through actin-binding proteins [40]. Taken together, the transcriptional pattern is consistent with an action at the filament-to-monomer balance and at its upstream regulators, although the present experiments do not measure filament and monomer pools directly.

The intestinal context adds a further dimension to the mechanism. Cofilin-1 and villin are junctional and apical regulators as well as actin-binding proteins, and cofilin-1 activity is required for normal junctional integrity and for the mucosal inflammatory response [3,41]. A preparation that raises *VIL1*, *CFL1* and *TMSB4X* transcription therefore acts on transcripts at the intersection of cytoskeletal architecture, barrier function and epithelial repair [4,42], which places the oyster endogenous peptide preparation among the luminal signals that the intestinal epithelium integrates at the level of its structural gene programme.

### 4.6. Food Relevance and Scope

The oyster is consumed raw and whole in many coastal regions, so a peptide that its tissue contains at the moment of consumption reaches the intestinal lumen without having passed through a processing step [22]. The measured yield of 2.41 mg of peptide per gram of wet soft tissues establishes the size of the endogenous peptide pool of the edible fraction, and concentrations of 0.1 to 2.0 mg/mL of that preparation change the cytoskeletal gene programme of a human intestinal epithelial cell line. The preparation and the monolayer therefore define a direct relationship between the peptide content of the edible tissue of this species and a human epithelial gene programme.

The plasma peptidome defines the peptide class that the animal’s own proteolytic system produces: its charge profile, its precursor preference and its size range. Every documented member of that population lies between 0.93 and 4.38 kDa, inside the 10 kDa window used for the tissue preparation, and 87.7% of it lies below 3 kDa, the size class that accounts for 71.2% of the tissue preparation. The two datasets come from different compartments by design. The published peptidome was obtained from hemolymph plasma [22], the circulating body fluid that serves as the immune compartment of molluscs and the compartment in which the antimicrobial effectors that the original study set out to characterise are found. The preparation used here was made from the soft body, which is the tissue that is eaten. Both compartments carry the endogenous peptide class that the animal’s own proteolytic system produces, and the two datasets therefore describe one peptide class in one species. The preparation was characterised by yield, size class, residual protein and endotoxin content, the attributes that determine what the monolayer encounters.

Shellfish remain a major allergen source and the knowledge gaps around shellfish allergy are substantial [77]. The peptide preparation used here is chemically distinct from the intact allergenic proteins of the tissue, which places its immunochemical behaviour in a different category from shellfish allergenicity.

### 4.7. Limitations and Future Perspectives

Several features of this work define the boundaries within which the findings should be read, and each of them indicates a specific next step. The experiments were performed in a single in vitro model, the differentiated Caco-2 monolayer, and therefore describe an epithelial response and not an effect in an intact organism. Three independent experiments were performed, each with a different cell passage and a different batch of the preparation; this design captures between-batch and between-passage variability but is a small number of biological replicates, and the effect sizes reported here should be read together with the confidence intervals rather than as precise estimates of magnitude.

The preparation was characterised by the attributes that determine what the monolayer encounters—yield, size class, residual protein and endotoxin content—and direct determination of its peptide composition is the step that follows. The peptidome that is analysed in this study was obtained from hemolymph plasma, whereas the preparation applied to the cells was made from the soft body. The two compartments are different by design, and the plasma peptidome is used here to define the charge profile, precursor preference and size range of the endogenous peptide class that this species produces; the re-analysis does not establish that the soft-tissue preparation contains the same peptides. Direct characterisation of the preparation by LC–MS/MS and a comparison with the deposited plasma peptidome are the next steps, and the present findings identify the anionic fraction as the class to characterise first.

The mechanistic interpretation rests on transcript abundance. The nine genes measured here are *SRF* targets and actin-monomer-binding genes, and the concentration dependence, the time course and the abolition of the response by proteolytic digestion together define a transcriptional signature. Measurement of MRTF-A localisation, of *SRF* activity and of the filament-to-monomer ratio, and the use of pathway inhibitors would test the mechanism directly, and the transcripts that responded here provide the readout against which such experiments can be designed.

Protein-level confirmation is the step that follows from these data. Transcript abundance and protein abundance are not equivalent, and the functional consequence of the transcriptional change belongs to the same next stage: barrier function was monitored before treatment, and paracellular permeability provides the complementary readout. Western blotting or immunofluorescence for the encoded proteins, and measurement of TEER and paracellular flux after treatment, would establish whether the transcriptional response is accompanied by a functional change in the monolayer. The individual responsible peptides remain to be identified, and fractionation of the preparation by charge and by size, followed by the same transcriptional readout, is the straightforward route to that identification and the experiment that would test whether the anionic class carries the activity.

Finally, the results describe an interaction at the apical surface of an epithelial monolayer. Whether the peptides survive gastrointestinal digestion, cross the epithelium, and reach concentrations at the epithelial surface comparable to those used here requires digestion models and bioavailability studies, and any extrapolation to human consumption depends on those measurements. The present work establishes the transcriptional response and the peptide dependence of that response, and it defines the experiments that follow.

## 5. Conclusions

An endogenous peptide preparation from oyster soft tissues, made without any added protease, changes the expression of the *SRF*-dependent cytoskeletal gene programme in differentiated human intestinal epithelial cells. The effect is concentration-dependent, present within 6 h, accompanied by concordant changes in transcripts encoding actin-monomer-binding proteins and abolished by proteolytic digestion of the preparation. In the same species, the endogenous plasma peptidome is anionically skewed, with 47.1% of peptides carrying a net charge of −1 or lower and only 8.4% carrying +2 or above, and the skew is invariant to infection status. The activity therefore resides in the endogenous peptide population rather than in the non-peptide material of the preparation. The individual responsible peptides remain to be identified, and the anionically skewed majority of this peptidome is the class that warrants direct investigation. Charge-based selection retains less than one tenth of this peptidome and sets aside the 47.1% that forms its anionic majority. Identifying the active peptides within that fraction and confirming their effects in functional and in vivo studies are the next steps.

## Figures and Tables

**Table 1:** qPCR primers, amplification characteristics and reference-gene stability.

Gene	Role	NCBI Accession	Forward Primer (5′→3′)	Reverse Primer (5′→3′)	Amplicon (bp)	Efficiency (%)	R^2^	Cq Range ^a^
*MYH9*	Primary target (*SRF* target)	NM_002473.6	GCACTGGTGAATCTGGAGCT	ATTCTTCACGGTCTTGGCGT	173	100.5	0.998	22.6–24.1
*ACTN1*	Primary target (*SRF* target)	NM_001130005.2	TTGACCGGAAGAAGACAGGC	GGGGTCCACAATGCTCATGA	116	97.6	0.997	23.2–24.8
*VIL1*	Primary target (*SRF* target)	NM_007127.3	GCAAGAGGAACGTGGTAGCT	ATGCCCCTGAGTCTCTCCAT	142	102.7	0.999	21.4–22.9
*CCN1*	Primary target (*SRF* target)	NM_001554.5	ATCAAGGACCCCATGGAGGA	CAAAAACAGGGAGCCGCTTC	139	96.1	0.996	25.1–26.7
*CFL1*	Secondary (actin-binding)	NM_005507.3	ATAAGGACTGCCGCTATGCC	CGTCCTTGGAGCTGGCATAA	138	98.8	0.998	19.8–21.2
*PFN1*	Secondary (actin-binding)	NM_005022.4	GTTCGTCAACATCACGCCAG	AAATTCCCCATCCTGCAGCA	136	101.3	0.999	19.5–20.9
*TMSB4X*	Secondary (actin-binding)	NM_021109.4	GCTTTTCCTCCGCAACCATG	CACGCCTCATTACGATTCGC	161	94.2	0.997	18.7–20.0
*SRF*	Secondary (pathway core)	NM_003131.4	TGCTGAATGCCTTCTCCCAG	ATCTGGTGGAGCTGAACTGC	145	104.4	0.996	25.4–27.0
*MRTFA*	Secondary (pathway core)	NM_020831.6	AGTACGCAGCCTCTCCACTA	AGCTCTGCCACCTTCATGTC	144	92.3	0.995	26.3–27.9
*B2M*	Reference gene (selected)	NM_004048.4	AGATGAGTATGCCTGCCGTG	TGCGGCATCTTCAAACCTCC	106	99.7	0.999	20.1–21.4
*HPRT1*	Reference gene (selected)	NM_000194.3	CCTGGCGTCGTGATTAGTGA	GGCCTCCCATCTCCTTCATC	163	98.4	0.998	23.1–24.3
*RPLP0*	Reference candidate	NM_001002.4	CTGGAGAAACTGCTGCCTCA	GCACCTTATTGGCCAGCAAC	100	96.8	0.998	20.6–21.8
*GAPDH*	Evaluated, not selected	NM_002046.7	GTCAAGGCTGAGAACGGGAA	AAATGAGCCCCAGCCTTCTC	158	101.8	0.997	18.2–19.4
*ACTB*	Evaluated, not selected	NM_001101.5	CTTCCTTCCTGGGCATGGAG	AATGCCAGGGTACATGGTGG	137	103.1	0.996	16.9–18.3

*B2M* and *HPRT1* were selected as reference genes (lowest geNorm M values; pairwise variation V < 0.15). ^a^ Cq ranges are the mean of three independent experiments and reflect the relative abundance of each transcript. Primer pairs were designed in silico with primer3 (v2.3.1) against NCBI RefSeq mRNA templates; in total, 12 of 14 pairs have at least one primer spanning an exon–exon junction so that genomic DNA is not amplified. Specificity was checked by BLAST (National Center for Biotechnology Information, Bethesda, MD, USA) against human RefSeq RNA. Standard curve: five ten-foldserial dilutions of pooled cDNA, three technical replicates per point. Efficiency (%) = (10^(−1/slope) − 1) × 100. Acceptance: 90–110%, R^2^ ≥ 0.99. Melt-curve analysis was performed from 65 to 95 °C; a single peak was accepted as specific amplification. No-template controls must show no amplification or Cq > 38. M values were calculated with geNorm from Cq matrices of all samples, including every candidate reference gene. M < 0.5 was accepted; M < 0.25 is excellent. GAPDH and ACTB were evaluated but not selected: transcript levels of both were affected by the treatments, which violates the assumption of a stably expressed reference gene.

**Table 2:** Anionic endogenous peptides of the oyster plasma peptidome (net charge ≤ −1 at pH 7; *n* = 157), the twenty most abundant by label-free intensity.

Peptide ID	Sequence	Length (aa)	Monoisotopic Mass (Da)	Net Charge (pH 7)	GRAVY	Precursor Protein	Actin Family	Mean Intensity, Unchallenged	Mean Intensity, Challenged
AP001	DLHQSELNVL	10	1166.6	−1.9	−0.24	EVMG016305.1	no	0	3,966,938,667
AP002	VVDNGSGMCKAGFAGDDAPRAVFPS	25	2467.1	−1	0.04	EVMG003590.1	yes	0	3,069,700,000
AP003	LVIDNGSGMCKAGFAGDDAPRAVFPS	26	2594.2	−1	0.2	EVMG000194.1	yes	0	2,477,300,000
AP004	LVVDNGSGMCKAGFAGDDAPRAVFPS	26	2580.2	−1	0.18	EVMG003590.1	yes	0	2,299,523,333
AP005	LEEMREDILKQMQITEEL	18	2247.1	−4	−0.72	EVMG002881.1_EVMG004543.1	no	0	2,262,076,667
AP006	VIDNGSGMCKAGFAGDDAPRAVFPS	25	2481.1	−1	0.05	EVMG000194.1	yes	0	2,029,846,667
AP007	VVDNGSGMCKAGFAGDDAPRA	21	2036.9	−1	−0.17	EVMG003590.1	yes	0	1,503,720,000
AP008	YNELRVAPEEHPVLL	15	1777.9	−1.9	−0.31	EVMG003590.1	yes	0	850,956,667
AP009	LVIDNGSGMCKAGFAGDDAPRA	22	2164	−1	0.02	EVMG000194.1	yes	0	834,765,000
AP010	NELRVAPEEHPVLL	14	1614.9	−1.9	−0.24	EVMG003590.1	yes	0	818,811,667
AP011	FEDIAVALEKNYTLKEMPAPICDAVNALK	29	3205.6	−2	0.12	EVMG000396.1	no	217,715,000	651,716,667
AP012	VVDNGSGMCKAGFAGDDAPR	20	1965.9	−1	−0.27	EVMG003590.1	yes	0	625,236,167
AP013	NELRVAPEEHPVL	13	1501.8	−1.9	−0.55	EVMG003590.1	yes	0	616,350,000
AP014	VIDNGSGMCKAGFAGDDAPRA	21	2050.9	−1	−0.16	EVMG000194.1	yes	0	613,386,667
AP015	SIQESTQLLEVLQ	13	1486.8	−2	0.02	EVMG005745.1	no	0	603,433,333
AP016	LVIDNGSGMCKAGFAGDDAPR	21	2093	−1	−0.06	EVMG000194.1	yes	0	569,428,333
AP017	LVVDNGSGMCKAGFAGDDAPR	21	2079	−1	−0.08	EVMG003590.1	yes	0	561,073,333
AP018	KEVMLQIEEFPEAKVEKFGAMFLDII	26	3053.6	−3	0.27	EVMG008490.1	no	0	543,216,667
AP019	YNELRVAPEEHPVL	14	1664.9	−1.9	−0.6	EVMG003590.1	yes	0	541,560,000
AP020	LVVDNGSGMCKAGFAGDDAPRA	22	2150	−1	0.01	EVMG003590.1	yes	0	536,825,000

Sequences are from the published plasma peptidome of *Crassostrea hongkongensis* (ProteomeXchange PXD025247); the tissue preparation used for the cell experiments was made from the soft tissue of the same species (Section 2.2). Net charge was calculated as (K + R + 0.1 × H) − (D + E); histidine is weighted at 0.1 to approximate its partial protonation at pH 7. GRAVY, grand average of hydropathicity (Kyte–Doolittle). Intensities are label-free MaxQuant values, means of three biological samples. An actin-family assignment was made by sequence: a reference-proteome entry of 350–400 residues containing the actin signature motif DNGSGMCKAGFAGDDAPR. Five such entries are present in the deposited proteome. Of the 333 peptides in the analysis set, 157 (47.1%) are in this charge class and 28 (8.4%) carry a net charge of +2 or above. The cationic minority is the class from which the six up-regulated peptides were selected for antimicrobial testing by the original authors. Peptides are ordered by mean label-free intensity in the challenged samples; the twenty listed are the twenty highest-ranked members of this charge class in the deposited dataset.

**Table 3:** Characterisation of the endogenous peptide preparation from *Crassostrea hongkongensis* soft tissues.

Batch ID ^a^	Tissue Input (g wet wt)	Total Peptide Yield (mg/g of Wet Tissues) ^b^	<3 kDa Fraction (%)	3–10 kDa Fraction (%)	Endotoxin (EU/µg Peptide) ^c^	Residual Protein (µg/mg Peptide) ^d^
P1	120	2.41 ± 0.18	71.3 ± 2.1	28.7 ± 2.1	0.043	21.4
P2	125	2.18 ± 0.14	68.9 ± 1.8	31.1 ± 1.8	0.061	26.8
P3	118	2.63 ± 0.21	73.5 ± 2.4	26.5 ± 2.4	0.038	18.9
Mean ± SD	121.0 ± 3.6	2.41 ± 0.23	71.2 ± 2.3	28.8 ± 2.3	0.047 ± 0.012	22.4 ± 4.0

Values are mean ± SD unless otherwise stated. ^a^ Three independent preparations (independent sampling, homogenisation and ultrafiltration); each batch assayed in triplicate. Each preparation came from a separate collection of 20 animals. ^b^ Determined by bicinchoninic acid assay after sequential ultrafiltration (10 kDa then 3 kDa cut-off); fraction percentages are expressed relative to total peptide recovered. Homogenisation buffer was exchanged for phosphate-buffered saline by three cycles of ten-fold dilution and re-concentration across a 3 kDa membrane before lyophilisation, so the material applied to cells contained PBS rather than homogenisation buffer. ^c^ Determined by kinetic turbidimetric Limulus amebocyte lysate assay with a standard curve and a positive product control in duplicate wells. Batches above 0.1 EU/µg peptide would be re-processed by polymyxin B affinity chromatography before use; all batches used for cell work were below that threshold. ^d^ Determined by Tricine SDS-PAGE (16.5% separating gel) with densitometric quantification, expressed per mg of peptide. No exogenous protease was added at any step, so the preparation contains peptides produced by the animal’s own proteolytic system. A vehicle control (PBS carried through the identical ultrafiltration, desalting and lyophilisation train, without tissue) was prepared alongside each batch and used as the vehicle in all cell experiments.

## Data Availability

The mass-spectrometry data re-analysed in this study are publicly available at ProteomeXchange under accession PXD025247. Processed data tables and the source data underlying each figure are provided as Supplementary Material.

## Supplementary Materials

The following supporting information can be downloaded at https://www.mdpi.com/article/10.3390/foods15213775/s1, Table S1 (qPCR primers and the complete set of amplification and validation characteristics, including the parameters additional to those in Table 1); Figures S1–S3 (PDF); and processed data tables underlying every reported value. Figure S1. Reproducibility and quality attributes of the endogenous peptide preparation. (A) Peptide yield of three independent preparations determined by bicinchoninic acid assay (three technical replicates per batch); bars show mean ± SD. (B) Molecular-weight distribution of the recovered peptide determined by sequential ultrafiltration. (C) Endotoxin content measured by kinetic turbidimetric *Limulus* amebocyte lysate assay. (D) Residual protein determined by Tricine SDS-PAGE with densitometric quantification, expressed per milligramme of peptide. In (C,D) the shaded band marks the acceptance range applied to every batch used for cell culture and the dashed line its upper limit. Figure S2. Selection and stability of the reference genes. (A) geNorm M values of the five candidate reference genes calculated from the Cq matrices of all samples, with the M = 0.5 acceptance limit; *B2M* and *HPRT1* were selected and *GAPDH* and *ACTB* were rejected. (B) Pairwise variation V as a function of the number of reference genes, with the V = 0.15 acceptance limit. (C) Cq matrix of the candidate reference genes across the twelve samples, from which the M and V values were computed; cells are coloured by Cq, and the geNorm M value of each gene is given at the right. Figure S3. Validation of quantitative PCR. (A) Standard curves for representative primer pairs generated from five ten-fold serial dilutions of pooled cDNA; error bars represent three technical replicates. (B) Melt curves for the same primer pairs, showing a single peak in each case. (C) Amplification efficiency of all fourteen primer pairs; the shaded band marks the 90–110% acceptance range. Table S1. qPCR primers and the complete set of amplification and validation characteristics. The parameters additional to those shown in Table 1 are given here.

## Abbreviations

*ACTN1*, α-actinin-1; *B2M*, β2-microglobulin; *CCN1*, cellular communication network factor 1; *CFL1*, cofilin-1; *GAPDH*, glyceraldehyde-3-phosphate dehydrogenase; GRAVY, grand average of hydropathicity; *HPRT1*, hypoxanthine phosphoribosyltransferase 1; LAL, *Limulus* amebocyte lysate; LPS, lipopolysaccharide; MRTF, myocardin-related transcription factor; *MYH9*, non-muscle myosin heavy chain 9; *PFN1*, profilin-1; *SRF*, serum response factor; TEER, transepithelial electrical resistance; *TMSB4X*, thymosin β4; *VIL1*, villin-1.
