# Supplementary material for: An Endogenous Peptide Preparation from Oyster Soft Tissues Modulates the Cytoskeletal Gene Expression Programme of Human Intestinal Epithelial Cells
Source: Foods. 2026 Oct 22;15(21):3775. doi: 10.3390/foods15213775 (PMC13762098; doi:10.3390/foods15213775)
Supplement: Supplementary file 1 [file foods-15-03775-s001.zip › foods-4610089-supplementary/foods-4610089-supplementary-FigS1.pdf]

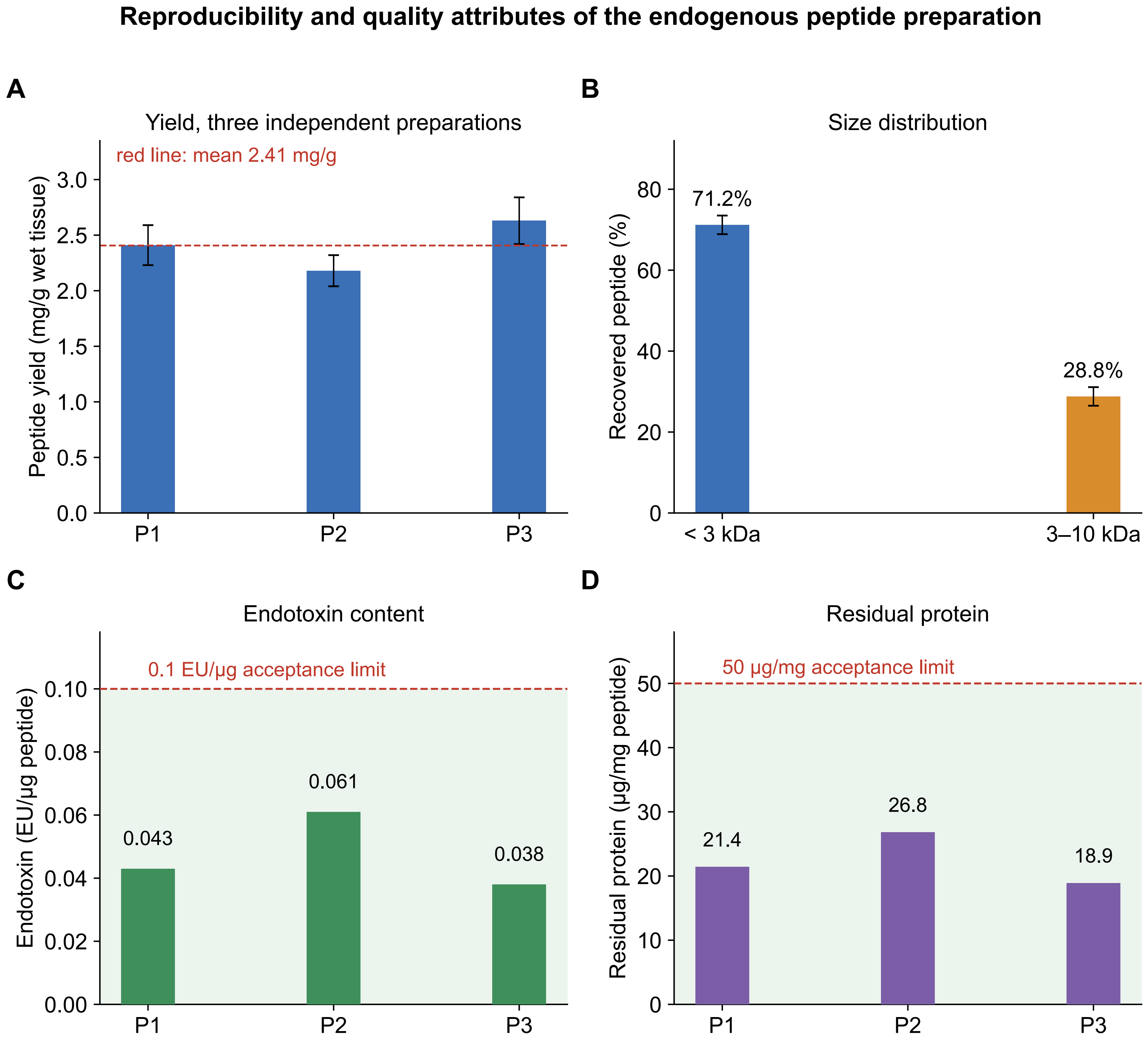
Reproducibility and quality attributes of the endogenous peptide preparation
A
Yield, three independent preparations
red line: mean 2.41 mg/g
Peptide yield (mg/g wet tissue)
0.0
0.5
1.0
1.5
2.0
2.5
3.0
P1
P2
P3
B
Size distribution
Recovered peptide (%)
0
20
40
60
80
71.2%
28.8%
< 3 kDa
3–10 kDa
C
Endotoxin content
0.1 EU/µg acceptance limit
Endotoxin (EU/µg peptide)
0.00
0.02
0.04
0.06
0.08
0.10
0.043
0.061
0.038
P1
P2
P3
D
Residual protein
50 µg/mg acceptance limit
Residual protein (µg/mg peptide)
0
10
20
30
40
50
21.4
26.8
18.9
P1
P2
P3
