# Supplementary material for: An Endogenous Peptide Preparation from Oyster Soft Tissues Modulates the Cytoskeletal Gene Expression Programme of Human Intestinal Epithelial Cells
Source: Foods. 2026 Oct 22;15(21):3775. doi: 10.3390/foods15213775 (PMC13762098; doi:10.3390/foods15213775)
Supplement: Supplementary file 1 [file foods-15-03775-s001.zip › foods-4610089-supplementary/foods-4610089-supplementary-FigS2.pdf]

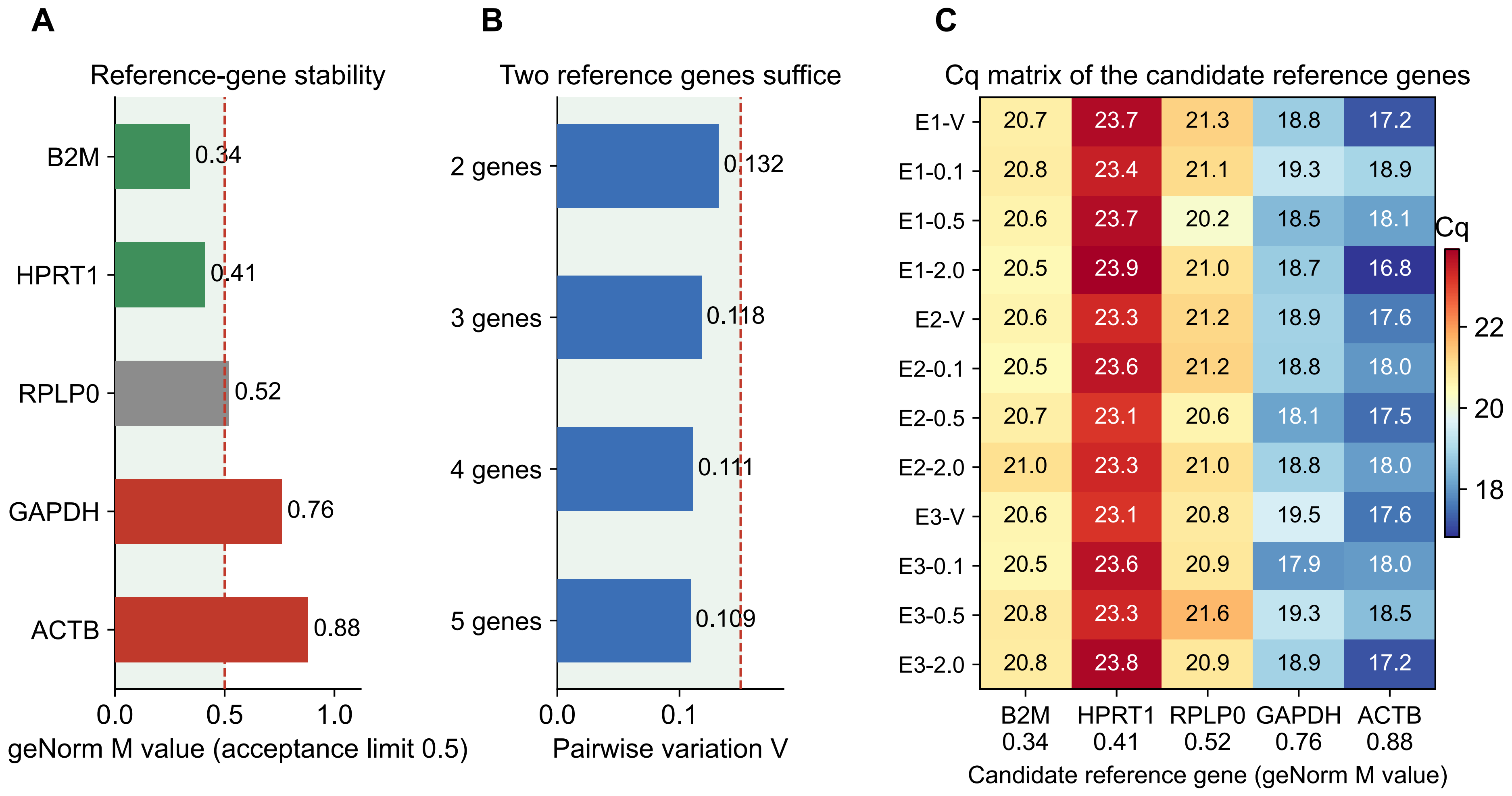
A
Reference-gene stability
B2M
HPRT1
RPLP0
GAPDH
ACTB
0.34
0.41
0.52
0.76
0.88
0.0
0.5
1.0
geNorm M value (acceptance limit 0.5)
B
Two reference genes suffice
2 genes
3 genes
4 genes
5 genes
0.132
0.118
0.111
0.109
0.0
0.1
Pairwise variation V
C
Cq matrix of the candidate reference genes
E1-V 20.7 23.7 21.3 18.8 17.2
E1-0.1 20.8 23.4 21.1 19.3 18.9
E1-0.5 20.6 23.7 20.2 18.5 18.1
E1-2.0 20.5 23.9 21.0 18.7 16.8
E2-V 20.6 23.3 21.2 18.9 17.6
E2-0.1 20.5 23.6 21.2 18.8 18.0
E2-0.5 20.7 23.1 20.6 18.1 17.5
E2-2.0 21.0 23.3 21.0 18.8 18.0
E3-V 20.6 23.1 20.8 19.5 17.6
E3-0.1 20.5 23.6 20.9 17.9 18.0
E3-0.5 20.8 23.3 21.6 19.3 18.5
E3-2.0 20.8 23.8 20.9 18.9 17.2
B2M 0.34
HPRT1 0.41
RPLP0 0.52
GAPDH 0.76
ACTB 0.88
Candidate reference gene (geNorm M value)
Cq
22
20
18
