# Supplementary material for: An Endogenous Peptide Preparation from Oyster Soft Tissues Modulates the Cytoskeletal Gene Expression Programme of Human Intestinal Epithelial Cells
Source: Foods. 2026 Oct 22;15(21):3775. doi: 10.3390/foods15213775 (PMC13762098; doi:10.3390/foods15213775)
Supplement: Supplementary file 1 [file foods-15-03775-s001.zip › foods-4610089-supplementary/foods-4610089-supplementary-FigS3.pdf]

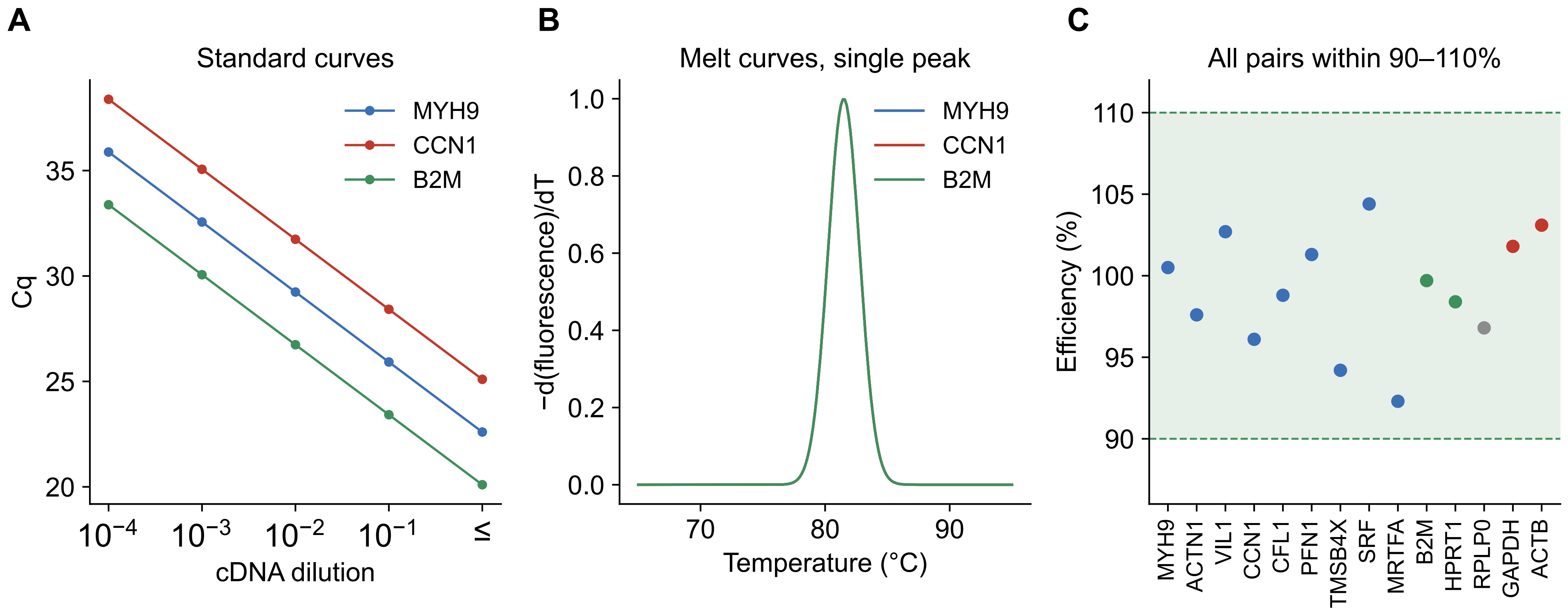

A
Standard curves
Cq
35
30
25
20
MYH9
CCN1
B2M
10^{-4}
10^{-3}
10^{-2}
10^{-1}
≤
cDNA dilution
B
Melt curves, single peak
−d(fluorescence)/dT
1.0
0.8
0.6
0.4
0.2
0.0
MYH9
CCN1
B2M
70
80
90
Temperature (°C)
C
All pairs within 90–110%
Efficiency (%)
110
105
100
95
90
MYH9
ACTN1
VIL1
CCN1
CFL1
PFN1
TMSB4X
SRF
MRTFA
B2M
HPRT1
RPLP0
GAPDH
ACTB
